# Modeling and rescue of defective blood–brain barrier function of induced brain microvascular endothelial cells from childhood cerebral adrenoleukodystrophy patients

**DOI:** 10.1186/s12987-018-0094-5

**Published:** 2018-04-04

**Authors:** Catherine A. A. Lee, Hannah S. Seo, Anibal G. Armien, Frank S. Bates, Jakub Tolar, Samira M. Azarin

**Affiliations:** 1 Department of Genetics and Cell Development, University of Minnesota, Minneapolis, MN 55455 USA; 2 Department of Chemical Engineering and Materials Science, University of Minnesota, Minneapolis, MN 55455 USA; 3 Ultrastructural Pathology Unit, Veterinary Diagnostic Laboratory, College of Veterinary Medicine, University of Minnesota, St. Paul, MN 55108 USA; 4 Department of Pediatrics, University of Minnesota, Minneapolis, MN 55455 USA

**Keywords:** Adrenoleukodystrophy, In vitro human blood–brain barrier (BBB) model, Brain microvascular endothelial cells, Trans-endothelial electrical resistance, Human induced pluripotent stem cells (hiPSC), Amphiphilic block copolymers

## Abstract

**Background:**

X-linked adrenoleukodystrophy (X-ALD) is caused by mutations in the *ABCD1* gene. 40% of X-ALD patients will convert to the deadly childhood cerebral form (ccALD) characterized by increased permeability of the brain endothelium that constitutes the blood–brain barrier (BBB). Mutation information and molecular markers investigated to date are not predictive of conversion. Prior reports have focused on toxic metabolic byproducts and reactive oxygen species as instigators of cerebral inflammation and subsequent immune cell invasion leading to BBB breakdown. This study focuses on the BBB itself and evaluates differences in brain endothelium integrity using cells from ccALD patients and wild-type (WT) controls.

**Methods:**

The blood–brain barrier of ccALD patients and WT controls was modeled using directed differentiation of induced pluripotent stem cells (iPSCs) into induced brain microvascular endothelial cells (iBMECs). Immunocytochemistry and PCR confirmed characteristic expression of brain microvascular endothelial cell (BMEC) markers. Barrier properties of iBMECs were measured via trans-endothelial electrical resistance (TEER), sodium fluorescein permeability, and frayed junction analysis. Electron microscopy and RNA-seq were used to further characterize disease-specific differences. Oil-Red-O staining was used to quantify differences in lipid accumulation. To evaluate whether treatment with block copolymers of poly(ethylene oxide) and poly(propylene oxide) (PEO–PPO) could mitigate defective properties, ccALD-iBMECs were treated with PEO–PPO block copolymers and their barrier properties and lipid accumulation levels were quantified.

**Results:**

iBMECs from patients with ccALD had significantly decreased TEER (2592 ± 110 Ω cm^2^) compared to WT controls (5001 ± 172 Ω cm^2^). They also accumulated lipid droplets to a greater extent than WT-iBMECs. Upon treatment with a PEO–PPO diblock copolymer during the differentiation process, an increase in TEER and a reduction in lipid accumulation were observed for the polymer treated ccALD-iBMECs compared to untreated controls.

**Conclusions:**

The finding that BBB integrity is decreased in ccALD and can be rescued with block copolymers opens the door for the discovery of BBB-specific molecular markers that can indicate the onset of ccALD and has therapeutic implications for preventing the conversion to ccALD.

**Electronic supplementary material:**

The online version of this article (10.1186/s12987-018-0094-5) contains supplementary material, which is available to authorized users.

## Background

The molecular mechanisms responsible for the onset and progression of childhood cerebral adrenoleukodystrophy (ccALD) remain poorly understood. ccALD is one form of X-linked adrenoleukodystrophy (X-ALD), an inherited metabolic storage disorder affecting 1 in 17,000 individuals [1]. X-ALD is caused by mutations in the *ABCD1* gene which codes for the ABCD1 protein [2]. ABCD1 is a peroxisomal transporter protein responsible for transporting very long-chain fatty acids (VLCFAs) from the cytosol into the peroxisome for subsequent beta-oxidation [3, 4]. Mutation type and location are not predictive of phenotype, as the same *ABCD1* mutation can lead to clinically distinct phenotypes [5–9]. A more frequent and less severe phenotype, adrenomyeloneuropathy (AMN), presents with demyelination in the long tracts of the spinal cord and progressive axonopathy, usually around the third or fourth decade of life. Heterozygous females will develop similar symptoms by age 60 [10–12]. ccALD, the most rapidly progressing phenotype, occurs in boys ages 2–12 and is characterized by sudden inflammatory demyelination in the brain and death within a few years [13, 14]. ccALD affects about 40% of males with an *ABCD1* mutation [15, 16]. MRI observation of gadolinium enhancement in the brain remains the only method to detect this progression [17–21]. Infections or head trauma have been described as initiators of the conversion from AMN to ccALD, but typically no extrinsic factor can be identified [22–24]. Current treatment for ccALD includes hematopoietic cell transplant (HCT), but this must be performed at the earliest stages of the disease [12, 14, 25, 26].

Much attention has focused on VLCFAs in the search for alternative treatments. While the accumulation of VLCFAs appears to directly contribute to symptoms of AMN, how VLCFAs contribute to the onset or progression of ccALD is unclear [27, 28]. VLCFAs accumulate in many tissue types in X-ALD patients, but this accumulation is not predictive of clinical phenotype [29, 30]. Furthermore, dietary regimens or treatments aimed at reducing the accumulation of VLCFAs (e.g. “Lorenzo’s oil”) cannot prevent ccALD onset [31–33], just as immunosuppression cannot prevent the cerebral inflammation seen during ccALD progression [34, 35]. Other biomarkers have been investigated for their potential correlation with ccALD conversion including mitochondrial defects, AMP-activated protein kinases, reactive oxygen species (ROS), and oxidative stress [15, 36–40]. Antioxidant activity levels of superoxide dismutase in blood plasma have been found to decrease prior to and during cerebral diagnosis [41]. Treatment with the antioxidant *N*-acetyl-l-cysteine improves survival of patients with advanced ccALD undergoing HCT [42], and oxidative stress levels decrease in patients after HCT [43]. A clinical trial testing a cocktail of antioxidants on patients with AMN has recently been completed though the results have yet to be published [44]. Identification at the molecular level of defects underlying the rapid BBB breakdown seen in ccALD would enable the development of strategies aimed at preventing the onset and progression of ccALD.

The initial blood–brain barrier (BBB) breakdown is thought to be mediated by immune cells (specifically T-cells and to some extent B-cells) translocating from the blood into the brain [45, 46]. Until recently, however, little attention has been paid to the brain endothelium constituting the BBB [47]. X-ALD lacks a suitable mouse model to study the BBB, as mice lacking ABCD1 only develop symptoms of AMN [48]. A human model of the BBB is difficult to obtain, as primary cells isolated from human brain biopsies are not readily available and tend to de-differentiate upon removal from the in vivo microenvironment [49]. Additionally, immortalized BMEC cell lines display poor barrier properties [50, 51]. To address these challenges, a system that enables modeling of the BBB through directed differentiation of human induced pluripotent stem cells (hiPSCs) into induced brain microvascular endothelial cells (iBMECs) was recently developed [52–55]. iBMECs from this system are readily renewable and have been shown to recapitulate important BMEC properties such as junctional protein expression, formation of a tight barrier with physiologically relevant trans-endothelial electrical resistance (TEER) (~ 5000 Ω cm^2^), and multidrug resistance protein efflux activity [54]. This system has been used to model the BBB of other neurological diseases such as Huntington’s [56] and to model bacterial interaction with the BBB [57]. Use of this system provides a unique opportunity to study the BBB of ccALD patients and to ask whether there are differences in barrier function compared to WT controls.

Additionally, this same system can be used to investigate potential therapeutic interventions to improve defects in barrier function. We hypothesized that treatment of diseased iBMECs with block copolymers of poly(ethylene oxide) (PEO) and poly(propylene oxide) (PPO) may improve barrier function. PEO–PPO–PEO triblock copolymers, called poloxamers or Pluronics, are widely used in biomedical applications due to their biocompatibility and amphiphilicity [58, 59]. Poloxamer 188 (P188; number average molar mass = 8.4 kg/mol and 80 wt% PEO) is approved for human use in certain applications [60] and has been demonstrated to provide cell membrane stabilization for a panoply of cell and tissue types under various stresses. P188 has been shown to be effective in ameliorating the effects of electropermeabilized skeletal muscle cells in rats [61], skeletal muscle cell necrosis [62, 63], dystrophic heart failure in mice [64], mechanical stress of dystrophic skeletal muscle in mice [65], damaged neuron-like cells in vitro [66, 67], injured primary neurons [68], and acute injury to the BBB in vivo [69–71]. Moreover, we recently found that a diblock analog of P188, a PEO–PPO diblock copolymer with one-half the composition and size of P188, can also protect model lipid membranes [72]. PEO–PPO diblock copolymers offer an opportunity to tune the end group on the PPO block, which recent work suggests to be an important molecular parameter in the ability of a PEO–PPO copolymer to confer protection [73]. A systematic in vitro screening of PEO–PPO diblock copolymers identified the polymer E_182_P_16_*t* (number average molar mass = 9 kg/mol and 90 wt% PEO; numerical subscripts indicate number of repeat units), which has a hydrophobic *tert*-butyl (*t*) end group on the PPO block, to be the most efficacious in stabilizing myoblasts under hypo-osmotic stress and isotonic recovery [74]. Thus, in addition to the commonly used Poloxamer 188, we hypothesized that E_182_P_16_*t* could also improve iBMEC function.

In this study, we used a previously established directed differentiation protocol to derive iBMECs from WT- and ccALD-iPSCs. This enabled us to model the BBB of ccALD patients and to examine potential differences in barrier function specific to ccALD. P188 and a PEO–PPO*t* diblock copolymer, E_182_P_16_*t*, were investigated for their potential to improve BMEC integrity. Testing of these two copolymers with this BBB model is a new avenue of investigation for X-ALD. Improvements in barrier function produced by amphiphilic block copolymers have implications for translation into a treatment for preventing the onset of ccALD by improving the BBB integrity of X-ALD patients. Translating the results from this study has the potential to reduce the number of individuals with X-ALD who develop deadly and rapidly progressive ccALD.

## Methods

### Derivation and culture of hiPSCs

Normal and ccALD iPSC lines (see Additional file 1: Table S1) were used [75, 76]. Cell lines were reprogrammed using retroviral gene delivery using the reprogramming factors OCT4, SOX2, KLF4, and c-MYC (Addgene) (WT1, WT2, ccALD1, ccALD2, ccALD3) or obtained from American Type Culture Collection (ATCC) (WT3 = ACS-1024). Cells were derived from somatic cells on irradiated MEF cultures and transferred to Matrigel (Corning) and E8 Medium (Thermo Fisher Scientific) or TeSR-E8 (STEMCELL Technologies) for additional feeder-free expansion and maintenance. All cell lines tested negative for Mycoplasma contamination via a MycoAlert™ Mycoplasma Detection Kit (Lonza). With the exception of the cell line obtained from ATCC, all cell lines were authenticated using genetic fingerprinting and were also found to be karyotypically normal.

### hiPSC differentiation to iBMECs

hiPSCs were differentiated according to Stebbins et al. [52]. On Day 8, cells were subcultured at a ratio of 1 well of a 6-well plate to 3 wells of a 12-well plate, 6 wells of a 24-well plate, Transwell filters (12 mm), or 11.4 wells of a µ-slide. When the cells reached confluence 48 h after subculture (Day 10), cells were utilized for permeability, efflux transporter, immunocytochemistry, RT-PCR, RNA-seq, and Oil-Red-O staining experiments using endothelial cell (EC) medium: human endothelial serum free medium (Thermo Fisher Scientific) with 1% platelet-poor plasma derived serum (Biomedical Technologies).

### Immunocytochemistry

iBMECs were subcultured onto µ-slides (Ibidi). 48 h post-subculture, cells were fixed in ice-cold 100% methanol (MilliporeSigma). Fixed iBMECs were blocked for 1 h at room temperature in a blocking buffer of PBS containing 10% normal goat serum (Thermo Fisher Scientific). Cells were incubated with primary antibodies diluted in blocking buffer overnight at 4 °C. After 3 washes with PBS for a minimum of 5 min per wash, cells were incubated with secondary antibody for 1 h in the dark at room temperature (see Additional file 1: Tables S2, S3). Cells were subsequently washed with PBS three times for a minimum of 5 min per wash and incubated with 4′,6-diamidino-2-pheny-lindoldihydrochloride (DAPI; Thermo Fisher Scientific) for 5 min to label nuclei. Cells were washed once with PBS for 5 min before imaging with an EVOS FL Auto Cell Imaging microscope.

### RT-PCR

Cells were differentiated as described above and detached with trypsin. Total RNA was extracted using an RNeasy Mini Kit (Qiagen) following the manufacturer’s protocol and quantified using a NanoDrop^®^ ND-1000. cDNA was generated from 1 µg of RNA using Omniscript reverse-transcriptase (Qiagen) and oligo-dT primers (Thermo Fisher Scientific). RT-PCR was performed using the GoTaq Green Master Mix (Promega) and PrimePCR primer sets (Bio-Rad) (see Additional file 1: Table S4). Glyceraldehyde-3-phosphate dehydrogenase (GAPDH) was used as the housekeeping gene. Gel electrophoresis of RT-PCR products with a 2% agarose gel was used to analyze transcript amplification.

### Trans-endothelial electrical resistance

iBMECs were seeded onto Transwell filters. TEER was measured daily starting 24 h after subculture utilizing the EVOM2 voltohmmeter with STX3 chopstick electrodes (World Precision Instruments). TEER was measured on an empty Transwell filter coated with collagen and fibronectin, and this value was subtracted from the TEER of the cell monolayer each time. TEER values were normalized by the surface area of the Transwell filter.

### Sodium fluorescein permeability

iBMECs were seeded onto Transwell filters. An empty Transwell filter coated with collagen and fibronectin was utilized to measure the permeability of the membrane. After a complete medium change, the cells were incubated at 37 °C for 1.5 h. TEER was measured before and after the medium change to confirm monolayer equilibration. Medium from the apical chamber was aspirated and replaced with EC medium containing 10 µM sodium fluorescein (MilliporeSigma). Every 30 min for 2 h, 150 µL aliquots were extracted from the basolateral chamber and replaced with 150 µL of fresh medium. At 2 h, a 150 µL sample was extracted from the apical chamber and then fluorescence was measured on a BioTek Synergy H1 multi-mode microplate reader at excitation of 485 nm and emission of 530 nm. Calculation of sodium fluorescein permeability was done following Stebbins et al. [52].

### Rhodamine 123 accumulation

Accumulation of rhodamine 123, a P-glycoprotein (P-gp) substrate, was measured in the absence and presence of a P-gp inhibitor cyclosporin A to quantify P-gp efflux potential. iBMECs were seeded onto 24-well plates. Cells were pre-incubated with or without 10 µM cyclosporin A (MilliporeSigma) in HBSS (Thermo Fisher Scientific) for 1 h at 37 °C. Next, all cells were incubated with 10 µM rhodamine 123 (MilliporeSigma) in HBSS for 2 h at 37 °C. Following the incubation steps, cells were lysed using RIPA buffer (MilliporeSigma) and fluorescence was measured on a BioTek Synergy H1 multi-mode microplate reader at excitation of 485 nm and emission of 530 nm. Unlysed cells from a parallel setup were dissociated with Accutase (Thermo Fisher Scientific) and counted using the Countess II to normalize the fluorescence on a per cell basis.

### Analysis of tight junction continuity

For quantitative analysis of iBMEC integrity, the percentage of cells expressing frayed tight junctions was counted using iBMECs immunolabeled for occludin. Cells were defined as having frayed tight junctions if any cell–cell contact point appeared discontinuous. A blinded analysis in which three different people each counted 15 separate frames and 12,530 total junctions was used to obtain a percentage of frayed tight junctions for both the ccALD- and WT-iBMECs.

### Electron microscopy

iBMECs were seeded onto 6-well plates. Two days after subculture, the cells were fixed with 1 mL of 2.5% glutaraldehyde (Electron Microscopy Sciences) in 0.1 M sodium cacodylate for 1 h at room temperature. Using cell lifters to detach the cells while preserving cell–cell junctions, cells were collected in microcentrifuge tubes and stored in fresh fixative solution for pelleting via centrifugation. Following 3 washes with 0.1 M sodium cacodylate buffer, cells were post-fixed with 1% osmium tetroxide (Electron Microscopy Sciences). Cells were dehydrated in acetone and subsequently embedded with Embed 812 resin (Electron Microscopy Sciences). A Leica UC6 Ultramicrotome (Leica Microsystems) was used to section the embedded samples. A JEM 1400 Plus transmission electron microscope (JEOL LTD) and AMT Capture Engine Version 7.00 (Advanced Microscopy Techniques Corp.) were used to analyze and image the samples.

### RNA-sequencing

Cells were differentiated as described above and detached with trypsin. Total RNA was isolated from WT- and ccALD-iBMECs using an RNeasy Mini Kit (Qiagen) following the manufacturer’s protocol. RNA with a RNA Integrity Number (RIN) score > 8 was used for library generation with the TruSeq Stranded mRNA Sample Preparation kit (Illumina). Paired-end 150 bp length reads were generated using an Illumina MiniSeq. Low quality bases were trimmed using Trimmomatic (enabled with the optional “--qualitycontrol” option and a 3 bp sliding-window trimming from the 3′ end requiring minimum Q16). The remaining reads were mapped to hg19 using Tophat2. The featureCounts program in the R SubRead package was used to generate a transcript abundance file for input into the R package edgeR to identify differentially expressed genes. Ingenuity Pathway Analysis [77] was used for network analysis and gene ontology [78, 79] for pathway analysis.

### Oil-Red-O staining and image analysis

iBMECs were seeded onto 12-well plates. Cells were fixed with 10% formalin for 20 min, subsequently dehydrated with 60% isopropanol, and incubated with Oil-Red-O (MilliporeSigma) for 10 min before being washed 4 times with deionized water. Images were captured using a Nikon Eclipse TS100 inverted light microscope connected to a Unitron Microscopes Lumenera^®^ Cameras AU-310-CMOS Infinity 1 camera. To measure the abundance of lipid droplets from the Oil-Red-O stained images, a custom MATLAB script was used to quantify the number and intensity of red pixels. Red pixels were defined on the HSV (hue, saturation, value) scale as having hue between 0.833 and 0.073, saturation between 0.300–1, and value between 0 and 1. The MATLAB Color Thresholder tool was used to mask as black any pixels not defined as red. Average intensity was measured by summing the value in the red channel of the RGB scale for each pixel, while the average number of pixels was calculated as the total number of red pixels.

### Diblock copolymer synthesis

E_182_P_16_*t* diblock copolymer was synthesized via ring-opening anionic polymerization following established techniques necessary for air and water free environments described elsewhere [72, 80, 81] with alumina column-dried tetrahydrofuran (THF) as the solvent. The PPO block was first synthesized at room temperature by initiation with potassium *tert*-butoxide (MilliporeSigma) in the presence of 18-crown-6 ether (MilliporeSigma) [82–84]. The reaction was carried out for 48 h, after which the reaction was terminated with excess acidic methanol (1:10 w/w% hydrochloric acid/methanol) to give *tert*-butoxy-terminated PPO chains. The PPO homopolymer was purified by iterative filtration, solvent removal, and dissolution in fresh THF. Subsequently, the hydroxyl terminated PPO was reinitiated with potassium naphthalenide, reacted with ethylene oxide for 20 h, and then terminated with excess acidic methanol. The resulting diblock copolymer was purified by iterative filtration, solvent removal, and dissolution in fresh THF. The final product was retrieved upon an additional purification step via dialysis.

### Polymer characterization

P188 (MilliporeSigma) and E_182_P_16_*t* were characterized via proton nuclear magnetic resonance spectroscopy (Bruker AX-400; deuterated chloroform as solvent) to determine compositions and/or number average molecular weight by end-group analysis. A size exclusion chromatograph (Waters) with a refractive index detector was used to obtain dispersity of the polymers. THF was utilized as the solvent, and the chromatograph was calibrated with polystyrene standards. The weight percent of PEO and dispersity of P188 were found to be 80 wt% and 1.06, respectively (number average molecular weight of P188 was provided by the manufacturer to be 8400 g/mol). The weight percent of PEO, number average molecular weight, and dispersity of E_182_P_16_*t* were determined to be 90 wt%, 8900 g/mol, and 1.07, respectively (see Additional file 1: Figure S1).

### Polymer treatment

Working solutions of polymers were prepared by dissolving P188 and E_182_P_16_*t* in 1X DPBS without magnesium or calcium (Thermo Fisher Scientific) to a concentration of 12 mM and sterilized via filtration. For polymer treatment during development, the polymers were added to the culture on Day 3 of the differentiation protocol. The polymer working solutions were diluted to a concentration of 0.5 mM or 1 mM in the culture medium, and the resulting solution was added to the culture during standard medium change. The control received medium with DPBS such that all conditions had the same volume of DPBS in the medium. After 24 h, the medium was changed in accordance with the differentiation protocol, effectively removing any excess polymers. On Day 8, the iBMECs were subcultured onto Transwells for TEER measurements or 12-well plates for Oil-Red-O staining. For polymer treatment post-differentiation, iBMECs were subcultured onto Transwells on Day 8; on Day 9, a small aliquot of the P188 or E_182_P_16_*t* working solutions was added to the apical chamber such that the final concentration of polymers in the apical chamber was 1 mM. A corresponding volume of DPBS was added to the control cells.

### Statistical analysis

Data are presented as mean ± standard error (SEM) with n defined in figure legends. *p*-values were determined using an unpaired Student’s *t*-test. Statistical analysis was performed using GraphPad Prism.

## Results

### Directed differentiation of WT- and ccALD-iPSCs into iBMECs

**Fig. 1:**
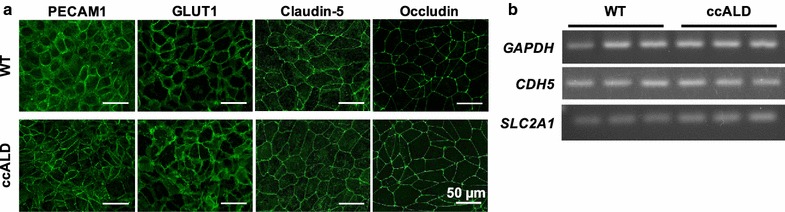
iBMECs express the requisite endothelial, tight junction, and BBB markers. **a** Representative immunocytochemistry (WT1 and ccALD2). iBMECs from ccALD patients and WT controls express PECAM1, GLUT1, claudin-5, and occludin. No qualitative difference was observed between the WT and ccALD-iBMECs. **b** RT-PCR (all WT and ccALD lines). iBMECs from ccALD patients and WT controls express *CDH5* (VE-cadherin) and *SLC2A1* (GLUT1)

A previously published protocol [52] was used to direct the differentiation of iPSCs into iBMECs from three clinically confirmed cases of ccALD and three WT controls. Immunofluorescence and RT-PCR demonstrated that both patient and control iBMECs expressed the requisite endothelial markers PECAM-1 and VE-cadherin (*CDH5*), the tight junction markers claudin-5 and occludin, and the BBB markers P-glycoprotein and GLUT-1 (*SLC2A1*) (Fig. 1a, b) (see Additional file 1: Figure S2). Since expression of *ABCB1,* which encodes the BMEC-specific efflux transporter P-gp, was decreased for one of the ccALD-iBMEC lines (see Additional file 1: Figure S3a), we employed a rhodamine 123 accumulation assay to check the P-gp efflux potential of the ccALD-iBMECs (see Additional file 1: Figure S3b). Normalized accumulation after inhibiting P-gp with cyclosporin A (CsA) was lower for the same iBMEC line in which we noticed the decreased *ABCB1* expression (102.7 ± 2.3) compared to the other ccALD-iBMEC lines (152.7 ± 23.4 and 167.1 ± 19.0) as well as the WT-iBMECs lines (258.2 ± 16.2, 220.8 ± 26.4, and 204.7 ± 20.2).

### ccALD-iBMECs have impaired barrier properties

**Fig. 2:**
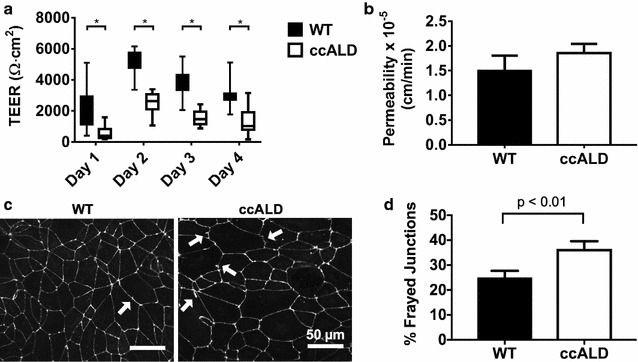
ccALD-iBMECs are functionally distinct from WT-iBMECs. **a** Trans-endothelial electrical resistance (TEER) is significantly decreased in the ccALD-iBMECs compared to the WT-iBMECs at all experimental time points. Data compiled from three independent experiments with nine biological replicates each (all iBMEC lines used) (n = 27). *p < 0.0001. **b** Passive transport as measured by sodium fluorescein permeability is slightly increased in the ccALD-iBMECs compared to WT-iBMECs. All iBMEC lines tested with three biological replicates each (n = 9). **c** Examples of frayed junctions indicated by white arrows on occludin immunolabeled images of WT1- and ccALD3-iBMECs. **d** Quantification of percent frayed junctions in WT1- and ccALD3-iBMECs indicates that WT-iBMECs have fewer frayed junctions than ccALD-iBMECs. Results of nine biological replicates with five technical replicates each shown (n = 45)

To investigate functional differences between the ccALD- and WT-iBMECs, we used trans-endothelial electrical resistance (TEER) to measure the barrier integrity of the iBMECs on Days 1–4 following subculture onto Transwell filters. At all days measured, we found a statistically significant difference (p < 0.0001) in TEER between the ccALD- and WT-iBMECs (peak TEER on Day 2 of measurement: 2592 ± 110 Ω cm^2^ compared to 5001 ± 172 Ω cm^2^ for the ccALD-iBMECs and WT-iBMECs, respectively) (Fig. 2a) (see Additional file 1: Figure S4 for TEER measurements for individual cell lines). Additionally, permeability of sodium fluorescein was measured to be 1.85 ± 0.19 × 10^−5^ cm/min for the ccALD-iBMECs and 1.50 ± 0.31 × 10^−5^ cm/min for the WT-iBMECs (Fig. 2b). The difference in permeability is not statistically significant despite the substantial difference (~ 2400 Ω cm^2^) in TEER between the WT- and ccALD-iBMECs; however, our results are consistent with previous studies that report sodium fluorescein permeability values on the order of 10^−5^ cm/min for BMECs with TEER greater than 2000 Ω cm^2^ [52, 53, 85, 86] and with reports that demonstrate that small molecule passive permeability does not correlate strongly with TEER above certain TEER thresholds [86–89]. To examine potential differences in tight junction organization, we employed a frayed junction analysis. The ccALD-iBMECs had more frayed junctions (p < 0.01) compared to the WT-iBMECs (37 ± 3% versus 25 ± 3%) (Fig. 2c, d). Overall, iBMECs from ccALD patients appear to form a less intact cellular barrier that permits increased passive transport of ions as well as small molecules. This defect in barrier integrity may result from mislocalization of tight junction proteins between cells.

### Lipid droplets accumulate in ccALD-iBMECs

**Fig. 3:**
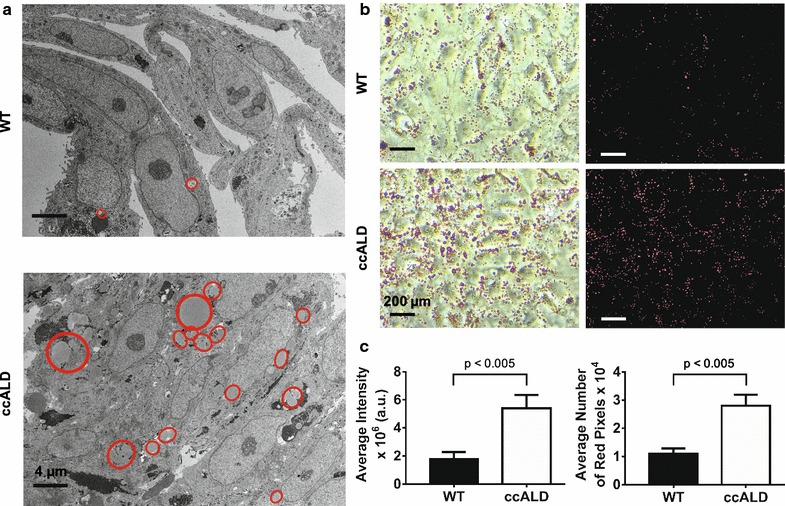
ccALD-iBMECs accumulate more lipid droplets than WT-iBMECs. **a** Comparison of transmission electron micrographs of WT1- and ccALD3-iBMECs show increased lipid droplet accumulation in ccALD-iBMECs. Lipid droplets outlined in red. **b** Representative images of Oil-Red-O stained WT3 and ccALD1-iBMECs. Raw images on left and masked images on right. **c** Quantification of intensity and number of red pixels in images of Oil-Red-O stained iBMECs indicate increased lipid droplet accumulation in ccALD-iBMECs compared to WT-iBMECs. Oil-Red-O staining images of all iBMEC lines were used for quantification using three biological replicates for each cell line (n = 9)

To assess any structural differences between the ccALD-iBMECs and WT controls, we performed transmission electron microscopy (TEM) of ccALD- and WT-iBMECs using cross-sections of fixed and pelleted cells. Numerous and large lipid droplets were present in the ccALD-iBMECs, with fewer and smaller lipid droplets in the WT-iBMECs (Fig. 3a) (see Additional file 1: Figure S5). To quantify the abundance of lipid droplets in the iBMECs, Oil-Red-O staining was used. This histological stain is specific to neutral lipids and does not stain the polarized phospholipids of the cell membrane [90]. Lipid droplets are stained bright red, and image analysis can be used to quantify either the amount (total number of red pixels) or intensity (redness of the red pixels) of red in micrographs. Quantification of the lipid deposition in ccALD- and WT-iBMECs revealed a significant increase (p < 0.005) in lipid abundance in the ccALD-iBMECs compared to the WT-iBMECs (Fig. 3b, c). The average intensity of red pixels in images of Oil-Red-O stained WT-iBMECs was calculated to be 1.8 ± 0.5 × 10^6^ compared to 5.4 ± 1.0 × 10^6^ for the ccALD-iBMECs. The average number of red pixels was calculated to be 1.1 ± 0.2 × 10^4^ for the WT-iBMECs and 2.8 ± 0.4 × 10^4^ for the ccALD-iBMECs (Fig. 3c). Very few lipid droplets were seen in the iPSCs and no statistical difference was observed between the ccALD-iPSCs and WT-iPSCs in the number of red pixels (22 ± 8 and 35 ± 11, respectively) or the intensity of red pixels (4.2 ± 2 × 10^3^ and 6.4 ± 2 × 10^3^, respectively) (see Additional file 1: Figure S6). VLCFA accumulation was not observed in the ccALD-iBMECs via TEM. The presence of an increased amount of lipid droplets in the ccALD-iBMECs compared to the WT-iBMECs that arises upon differentiation (i.e. is not present during the iPSC stage) is a difference that potentially contributes to the decreased barrier integrity of the ccALD-iBMECs.

### Transcriptome analysis indicates differences in Type I interferon activation and lipid metabolism pathways

**Fig. 4:**
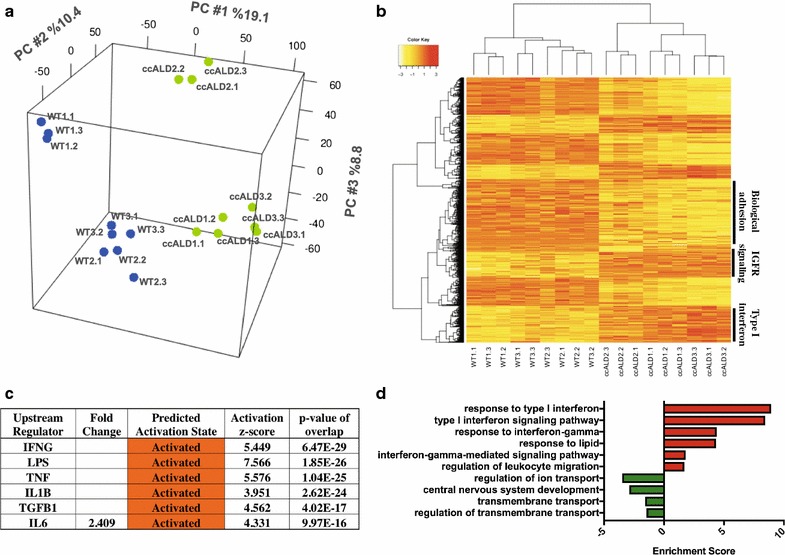
Transcriptome analysis indicates differences in Type I interferon activation and lipid metabolism pathways. **a** PCA mapping of log_2_ normalized read counts on global gene expression. The first three dimensions account for 38.3% of the total variance with grouping of individual WT- and ccALD-iBMEC replicates and separation of the experimental and control samples along PC1. **b** Heat map of DEG (n = 1381) on log_2_ normalized read counts. Cluster annotations are from gene ontology analysis. **c** IPA upstream regulator analysis of transcriptional regulators predicted by activation z-scores. p-values calculated by Fisher’s exact test using expected and observed genes overlapping with the WT versus ccALD DEGs and all genes regulated by each transcriptional regulator. **d** GO terms of pathways upregulated in ccALD in red with downregulated pathways in green. Data analyzed from three independent experiments with three biological replicates each (n = 9)

To further characterize differences between ccALD- and WT-iBMECs and to elucidate potential mechanisms for the decreased barrier integrity seen in the ccALD-iBMECs, we performed RNA-sequencing of three replicate differentiations for each of our ccALD- and WT-iBMEC lines. Principal component analysis (PCA) separated the WT-iBMECs from the ccALD-iBMECs along the first principal component (Fig. 4a). Hierarchical clustering of differentially expressed genes (DEGs) (2× fold change, false-discovery rate (FDR) < 0.05) and samples revealed a cluster of genes that were decreased in the ccALD-iBMECs involving the attachment of cells to each other including intracellular attachment between membrane regions (gene ontology (GO): 0022610), while Type I interferon-activated signaling (GO: 0060337) and insulin-like growth factor receptor signaling (GO: 0043568) pathways were increased in the ccALD-iBMECs (Fig. 4b). We used Ingenuity Pathway Analysis (IPA) to query upstream regulators of our DEGs. IPA builds a graph-based network from gene expression data and uses this information to predict upstream regulators. The z-score (calculated as the number of standard deviations from the mean of a normal distribution of activity edges using this graph-based network) represents the magnitude of bias in gene regulation that predicts the activity of specific upstream regulators. This analysis revealed upstream regulators involving TGFβ1 signaling, Type I interferon response, and other immune signaling signatures highly activated in the ccALD-iBMECs (IFNG, LPS, TNF, and TGFβ1 z-scores of 5.5, 7.6, 5.6, and 4.6 respectively) (Fig. 4c). GO analysis was performed on genes differentially expressed between the ccALD- and WT-iBMECs. This analysis calculates a p-value based on enrichment of genes in a particular GO annotation (− log_10_(p-value) reported as enrichment score for upregulated genes, log_10_(p-value) reported as enrichment score for downregulated genes). GO analysis on genes upregulated (increased activity) in ccALD-iBMECs indicated an increase in Type I interferon signaling (enrichment score 8.45) and response to lipid pathways (enrichment score 4.34). GO analysis on genes downregulated (decreased activity) in ccALD-iBMECs indicated a decrease in transmembrane and ion transport (enrichment scores − 1.58 and − 3.5, respectively) (Fig. 4d). The lipid pathway upregulation is consistent with both the primary ccALD phenotype and our TEM results, while Type I interferon signaling and other inflammatory pathways are secondary and could have many sources.

### Block copolymers reverse impaired barrier integrity and mitigate lipid accumulation

**Fig. 5:**
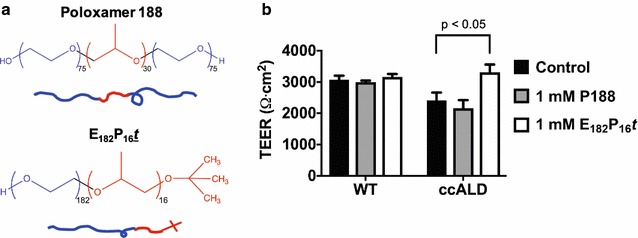
Diblock copolymer treatment rescues defective barrier function of ccALD-iBMECs. **a** Chemical structures of polymers utilized for treatment. Poloxamer 188 is a triblock copolymer of poly(ethylene oxide) (PEO) and poly(propylene oxide) (PPO); E_182_P_16_*t* is a diblock copolymer of PEO and PPO with a *tert*-butoxy end group on the PPO block. **b** Maximum TEER of WT1- and ccALD3-iBMECs treated with 1 mM of P188 or E_182_P_16_*t*. Treatment with 1 mM E_182_P_16_*t* resulted in improved ccALD-iBMEC barrier function. Data shown from four independent experiments with three biological replicates each (n = 12)

**Fig. 6:**
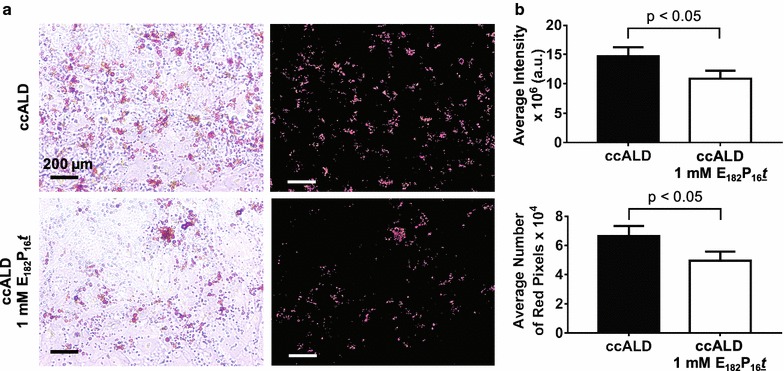
Diblock copolymer treatment decreases lipid droplet accumulation in ccALD-iBMECs. **a** Representative Oil-Red-O staining of untreated control ccALD3-iBMECs and 1 mM E_182_P_16_*t* treated ccALD3-iBMECs during development. Raw images shown on left and masked images on right. **b** Quantification of intensity and number of red pixels in Oil-Red-O stained images indicates decreased lipid droplet accumulation in ccALD-iBMECs treated with 1 mM E_182_P_16_*t* during development. Six biological replicates used for quantification (n = 6)

We next investigated whether polymer treatment could rescue the impaired barrier integrity of the ccALD-iBMECs. ccALD-iBMECs were treated with 1 mM of P188 or E_182_P_16_*t* at the end of the differentiation protocol (Day 9) or during development (Day 3) (see Fig. 5a for chemical structures of the polymers). Day 3 was chosen because the cells begin to express endothelial cell markers at this time point [55]. Polymer treatment with either P188 or E_182_P_16_*t* at the end of the differentiation protocol showed minimal effect on ccALD-iBMEC TEER (see Additional file 1: Figure S7a). However, we saw a significant effect (p < 0.05) when the ccALD-iBMECs were treated with the diblock copolymer (E_182_P_16_*t*) during development. The maximum TEER of the ccALD-iBMECs treated with E_182_P_16_*t* was 3316 ± 246 Ω cm^2^. This was higher than both the untreated and P188 treated ccALD-iBMECs (2409 ± 254 and 2162 ± 260 Ω cm^2^, respectively; Fig. 5b). The effect of dosage was investigated by treating the ccALD-iBMECs with 0.5 or 1 mM E_182_P_16_*t* on Day 3 of the differentiation protocol, and we observed a larger increase in TEER compared to the control when treated with 1 mM E_182_P_16_*t* than with 0.5 mM E_182_P_16_*t* (see Additional file 1: Figure S7b). Notably, the barrier function of the WT-iBMECs was unaffected by polymer treatment as the TEER of the untreated WT-iBMECs (3074 ± 127 Ω cm^2^) was not significantly different than that of WT-iBMECs treated with P188 (2998 ± 50 Ω cm^2^) or E_182_P_16_*t* (3165 ± 95 Ω cm^2^). Additionally, lipid droplet accumulation was decreased in ccALD-iBMECs treated with 1 mM E_182_P_16_*t* during development (Fig. 6). Quantification of Oil-Red-O staining images indicated a statistically significant (p < 0.05) decrease in ccALD-iBMECs treated with 1 mM E_182_P_16_*t* when compared to untreated ccALD-iBMECs. The average intensity of red pixels in images of Oil-Red-O stained ccALD-iBMECs treated with 1 mM E_182_P_16_*t* was 10.9 ± 1 × 10^6^ compared to 14.8 ± 1 × 10^6^ for the control. The average number of red pixels was calculated to be 4.9 ± 0.5 × 10^4^ for ccALD-iBMECs treated with 1 mM E_182_P_16_*t* and 6.7 ± 0.5 × 10^4^ for the control.

## Discussion

Our model of the BBB demonstrating that the barrier function is defective and lipid droplets accumulate in iBMECs from patients with ccALD opens the door to new therapeutic avenues aimed at maintaining the integrity of the BBB and preventing the onset of ccALD. In the present work, our findings indicate a significant improvement in barrier function and a decrease in lipid droplet accumulation when ccALD-iBMECs are treated during differentiation with a diblock copolymer with a hydrophobic *tert*-butoxy end group (E_182_P_16_*t*). No effect was seen when the ccALD-iBMECs were treated at the same time with P188 or when the ccALD-iBMECs were treated post differentiation with either polymer. Treatment of WT-iBMECs with either polymer during development did not improve barrier function. Response of the ccALD-iBMECs but not the WT-iBMECs to polymer treatment further highlights that there are fundamental differences between the ccALD- and WT-iBMECs.

Overall, our study demonstrates that one of the intrinsic defects in ccALD is with the integrity of the BMECs that constitute the BBB. These findings are in line with the study by Musolino et al. [47] in which they knocked down *ABCD1* in BMECs and saw mislocalization of the tight junction protein claudin-5. The presence of frayed or discontinuous junctions and its relation to barrier function has been noted in other systems as well. In an in vitro epithelium model, an induced opening of the barrier for drug delivery purposes was marked by both morphological changes in the connectivity of zonula occluden tight junction proteins and a decrease in TEER [91]. In our study of the brain endothelium, we went beyond qualitative observations of tight junction proteins and quantified the integrity of the barrier formed by the WT- and ccALD-iBMECs using TEER. By this metric, we found that the barrier integrity of the ccALD-iBMECs was decreased compared to WT-iBMECs. Musolino et al. also observed an increase in TGFβ1 expression connected to the mislocalization of claudin-5. Interestingly, our transcriptome analysis also indicated increased TGFβ1 activity in the ccALD-iBMECs that could be contributing to the decreased barrier function. With ccALD, in contrast to other demyelinating disorders such as multiple sclerosis, demyelination is thought to precede BBB breakdown. Thus, it is possible that an initial subtle loss in the ability of the BBB to restrict passive transport, as seen with the ccALD-iBMECs in our study, could cause immune cell infiltration and leakage that accelerates demyelination in a feedback loop that eventually results in complete BBB breakdown [29]. In this context, increased matrix metalloproteinases in the cerebral spinal fluid of ccALD patients could also be contributing to further breakdown of the BBB [38, 47]. Inherent decreased BBB integrity could also begin to explain why head trauma can initiate the onset of ccALD. The lack of genotype–phenotype correlation is not explained by our model; however, our finding of an inherent decrease in BMEC integrity in ccALD individuals could direct the search for additional environmental or genetic factors specific to the BBB that begin to explain why only a subset of individuals with an *ABCD1* mutation progress to ccALD.

While we did not observe by TEM the classic crystalline aggregates first observed in the adrenal cortex, testis, and white matter of ccALD patients, our finding of increased accumulation of non-pathological lipid droplets in ccALD-iBMECs is novel and warrants further investigation [92–94]. A study by Schluter et al. [95] showed that VLCFAs can trigger insulin desensitization characterized by oxidative stress and alteration of adipocytokine signaling pathways and chronic inflammation, culminating in changes similar to metabolic syndrome. Increased insulin-like growth factor receptor signaling in the ccALD-iBMECs indicated by our transcriptome analysis further hints at metabolic dysfunction as a factor contributing to the ccALD phenotype. Another study by van de Beek et al. [96] showed that exposure of X-ALD fibroblasts to VLCFAs resulted in endoplasmic reticulum stress correlated with an increase in lipid droplet deposition. Both studies suggest that VLCFA accumulation would precede lipid droplet accumulation. A key question that then arises is whether lipid droplet accumulation contributes to the decreased BBB integrity in the ccALD-iBMECs and whether targeting non-VLCFA lipids would have therapeutic relevance in that it could potentially rescue the decrease in BBB integrity of ccALD patients.

Using this system to model the BBB, we achieved physiological levels of TEER. One limitation of our study, however, is that we only investigated one cell type, BMECs. Adding other cell types involved in the neurovascular unit such as pericytes, astrocytes, and neurons to our model could further inform ccALD-specific defects of the BBB. Nevertheless, the differences we found modeling the BBB using iBMECs were significant. These differences included a decrease in barrier integrity as well as an increase in lipid accumulation. Both of these findings represent potential biomarkers for brain endothelium health of X-ALD patients and provide a new direction in the search for molecular markers that indicate ccALD onset. Combining the findings from the results of this study with antioxidant therapy currently in clinical trials could provide a much-needed alternative treatment for patients with AMN at risk of converting to ccALD.

With our BBB model, we investigated whether amphiphilic block copolymers can improve defects in barrier function as such polymers have been reported to be able to improve function of many cell and tissue types under various injuries. The application of the diblock copolymer E_182_P_16_*t* in addition to the widely used P188 was inspired by recent work within our group, which revealed E_182_P_16_*t* to be the most efficacious in stabilizing damaged myoblasts in vitro [74]. It is interesting and crucial to note that this enhanced efficacy of E_182_P_16_*t* compared to P188 is consistent with the results of Kim et al. although the cell types and form of damage are vastly different [74]. This raises many questions as to how the polymers interact with the cell and what cellular responses this interaction promotes. At present, the fundamental mechanism of interaction between the PEO–PPO diblock and triblock copolymers and the plasma membrane is far from conclusive. Lee et al. speculate that poloxamers insert partially or fully into the membrane after initially adsorbing onto the lipid bilayer depending on the hydrophobicity of the polymer and the incubation time [97, 98]. Enhanced efficacy with the presence of an additional hydrophobic *tert*-butoxy end group on the PPO block provides evidence for the “anchor and chain” mechanism, which proposes that the additional hydrophobic unit at the end of the PPO block provides an anchor in the lipid bilayer, resulting in more efficacious stabilization of the block copolymer in the lipid membrane [73, 74]. The aforementioned studies focus on the polymer interaction with the plasma membrane for stabilization, but the results of our work showing the decrease in lipid accumulation in ccALD-iBMECs upon treatment with E_182_P_16_*t* suggest a more complex cellular response that has yet to be fully explored.

As the breadth of applications continues to expand, there is a pressing need to elucidate the amphiphilic block copolymer-cell interaction mechanism in order to translate this action into a therapeutic solution. To this end, the in vitro disease model presented in this work could provide a platform for studying the mechanism of PEO–PPO block copolymer mediated recovery of cellular function. Furthermore, while most researchers have focused solely on P188, there is potential for the design of the polymer to further improve efficacy in restoring function to damaged cells as demonstrated in this work. Elucidating the mechanism of BMEC interaction with the PEO–PPO diblock copolymer will not only engender insight as to how the polymer restores function of ccALD-iBMECs but may also provide a deeper knowledge as to how the BBBs of ccALD patients are damaged compared to healthy individuals.

At present, there is no suitable in vivo model for X-ALD [48]. Nevertheless, as functional in vivo models are developed, the work presented here has the potential to be translated to in vivo studies. Treatment of ccALD-iBMECs with either P188 or E_182_P_16_*t* at the end of the differentiation protocol yielded a slight but non-significant increase in TEER. However, efficacy of polymer treatment at a later stage might be improved upon optimization of pharmacodynamics and pharmacokinetic variables. Furthermore, the superior efficacy of treatment with E_182_P_16_*t* when added earlier in the iBMEC differentiation process compared to at the end of the differentiation process suggests that the treatment could be applied at an early stage of BBB development to inhibit the onset and progression of ccALD.

Thus, clinical application might mean using the polymer as a preventative therapy, which requires pre-symptomatic diagnosis of X-ALD. Fortunately, high throughput screening of X-ALD is feasible and reliably identifies affected males [99]. Furthermore, in 2016 the US Department of Health and Human Services recommended that X-ALD be added to the recommended uniform screening panel for state newborn screening programs [100]. Testing of the 4 million infants born each year in the US is predicted to identify around 143 newborns with an *ABCD1* mutation. Early detection will lead to more timely intervention in the form of hematopoietic cell transplant (HCT), which is only advantageous in the early stages of the disease because cerebral inflammation can progress up to 18 months after transplant [25, 101]. Pioneering clinical trials involving the use of Lenti-D for autologous HCT are taking place at several centers around the US and promise to further reduce severe outcomes associated with allogeneic transplants (such as graft-versus-host disease) and to circumvent issues with finding HLA matched donors (currently, cord blood grafts are used when a suitable donor cannot be found) [102–104]. For those displaying neurological symptoms or MRI abnormalities indicating ccALD onset, the current standard of care for HCT involves fully myoablative chemotherapy, a highly toxic procedure. If a treatment that prevents the onset of ccALD were available, a newborn identified as having an *ABCD1* mutation given this treatment may never show symptoms of ccALD conversion and would not need to undergo HCT. In this study, we have shown that amphiphilic block copolymers are one such treatment with the potential to prevent the onset of ccALD and reduce the number of patients needing to undergo HCT.

## Conclusion

Modeling the BBB of ccALD patients using iPSC-derived BMECs indicates that ccALD patients form a less intact BBB. These results open the door for the discovery of brain endothelium-specific molecular markers indicative of the onset of ccALD and for the development of treatment strategies targeted at the brain endothelium that could reduce the number of X-ALD patients who progress to ccALD. One such treatment strategy that we have shown can rescue defective ccALD-iBMECs barrier integrity is PEO–PPO block copolymers. These results have therapeutic implications for preventing the onset of ccALD.

## Additional file

**Additional file 1: Table S1.** Information on induced pluripotent stem cell (iPSC) lines used in study. **Table S2.** Primary antibodies used for immunocytochemistry. **Table S3.** Secondary antibodies used for immunocytochemistry. **Table S4.** Primers used for RT-PCR. **Figure S1.** Polymer characterization data. **Figure S2.** Representative immunocytochemistry images of iBMEC lines not shown in main manuscript. **Figure S3.** P-glycoprotein (P-gp) expression and function. **Figure S4.** TEER measurements for individual cell lines. **Figure S5.** Additional representative TEM images. **Figure S6.** Oil-Red-O staining and quantification of WT and ccALD-iPSCs. **Figure S7.** Timing and dosage effect of polymer treatment.

## Abbreviations

X-ALD: X-linked adrenoleukodystrophy
ccALD: childhood cerebral adrenoleukodystrophy
BBB: blood–brain barrier
ROS: reactive oxygen species
WT: wild-type
iPSCs: induced pluripotent stem cells
iBMECs: induced brain microvascular endothelial cells
BMECs: brain microvascular endothelial cells
TEER: trans-endothelial electrical resistance
VLCFA: very long chain fatty acids
AMN: adrenomyeloneuropathy
HCT: hematopoietic cell transplant
hiPSCs: human induced pluripotent stem cells
PEO: poly(ethylene oxide)
PPO: poly(propylene oxide)
P188: Poloxamer 188
EC: endothelial cell
RIN: RNA Integrity Number
THF: tetrahydrofuran
P-gp: P-glycoprotein
CsA: cyclosporin A
TEM: transmission electron microscopy
PCA: principal component analysis
DEG: differentially expressed gene
FDR: false-discovery rate
GO: gene ontology
IPA: Ingenuity Pathway Analysis
