# Supplementary material for: Modeling and rescue of defective blood–brain barrier function of induced brain microvascular endothelial cells from childhood cerebral adrenoleukodystrophy patients
Source: Fluids Barriers CNS. 2018 Apr 4;15:9. doi: 10.1186/s12987-018-0094-5 (PMC5883398; doi:10.1186/s12987-018-0094-5)
Supplement: Supplementary file 1 — Additional file 1: Table S1. Information on induced pluripotent stem cell (iPSC) lines used in study. Table S2. Primary antibodies used for immunocytochemistry. Table S3. Secondary antibodies used for immunocytochemistry. Table S4. Primers used for RT-PCR. Figure S1. Polymer characterization data. Figure S2. Representative immunocytochemistry images of iBMEC lines not shown in main manuscript. Figure S3. P-glycoprotein (P-gp) expression and function. Figure S4. TEER measurements for individual cell lines. Figure S5. Additional representative TEM images. Figure S6. Oil-Red-O staining and quantification of WT and ccALD-iPSCs. Figure S7. Timing and dosage effect of polymer treatment. [file 12987_2018_94_MOESM1_ESM.docx]

**Supplementary Information**

**Modeling and rescue of defective blood-brain barrier function of induced brain microvascular endothelial cells from childhood cerebral adrenoleukodystrophy patients**

Catherine A.A. Lee, Hannah S. Seo, Anibal G. Armien, Frank S. Bates, Jakub Tolar, Samira M. Azarin

**Inventory of supplementary information**

Table S1

Table S2

Table S3

Table S4

Figure S1

Figure S2

Figure S3

Figure S4

Figure S5

Figure S6

Figure S7

**Table S1. Information on induced pluripotent stem cell (iPSC) lines used in study**

| **iPSC lines** | **Sex** | **Derived cell type** | **Delivery method** | **Reprogramming factors** |
| --- | --- | --- | --- | --- |
| ccALD1 | Male | Fibroblasts | Retrovirus | OCT4, SOX2, KLF4, c-MYC |
| ccALD2 | Male | Fibroblasts | Retrovirus | OCT4, SOX2, KLF4, c-MYC |
| ccALD3 | Male | Keratinocytes | Retrovirus | OCT4, SOX2, KLF4, c-MYC |
| WT1 | Female | Keratinocytes | Retrovirus | OCT4, SOX2, KLF4, c-MYC |
| WT2 | Male | Urine cells | Retrovirus | OCT4, SOX2, KLF4, c-MYC |
| WT3 | Male | CD34+ bone marrow cells | Sendai virus | OCT4, SOX2, KLF4, c-MYC |

**Table S2. Primary antibodies used for immunocytochemistry**

| **Target antigen** | **Antibody species** | **Vendor** | **Clone or product number** | **Dilution** |
| --- | --- | --- | --- | --- |
| PECAM-1 | Rabbit | ThermoFisher | RB10333P | 1:100 |
| GLUT-1 | Mouse | ThermoFisher | MS10637P1; clone SPM498, IgG2a | 1:100 |
| Occludin | Mouse | ThermoFisher | 331500; clone OC-3F10 | 1:200 |
| Claudin-5 | Mouse | ThermoFisher | 352500; clone 4C3C2 | 1:50 |

**Table S3. Secondary antibodies used for immunocytochemistry**

| **Species reactivity** | **Host** | **Conjugate** | **Vendor** | **Dilution** |
| --- | --- | --- | --- | --- |
| Mouse | Goat | Alexa Fluor 594 | ThermoFisher | 1:200 |
| Rabbit | Goat | Alexa Fluor 594 | ThermoFisher | 1:200 |

**Table S4. Primers used for RT-PCR**

| **Gene** | **Vendor** | **ID Number** |
| --- | --- | --- |
| *GAPDH* | BioRad | qHsaCED0038674 |
| *CDH5* | BioRad | qHsaCID0016288 |
| *SLC2A1* | BioRad | qHsaCID0022232 |
| *ABCB1* | BioRad | qHsaCID0020960 |

**
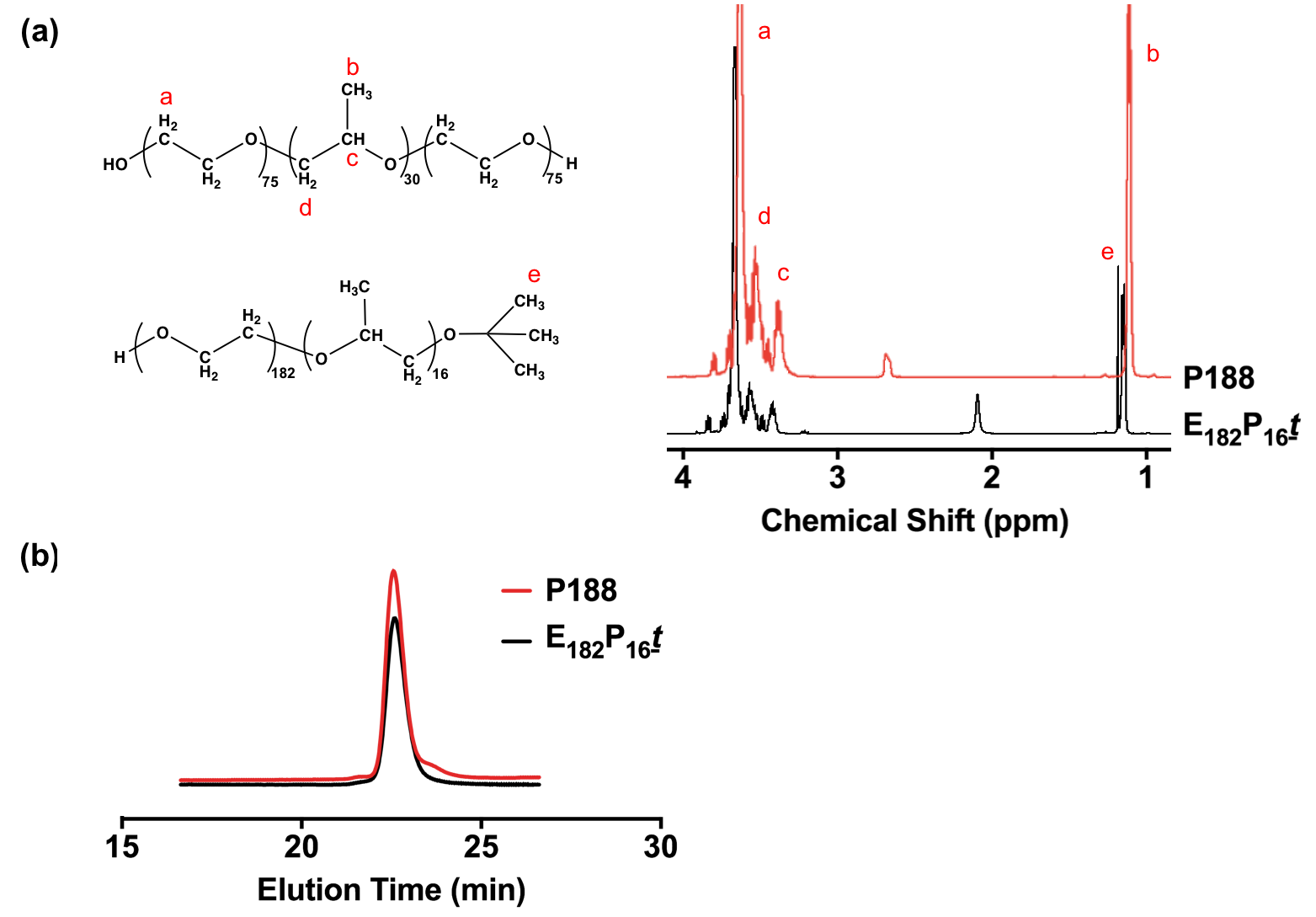
**

**Figure S1. Polymer characterization data.** (**a**) ^1^H-NMR spectra of P188 and E_182_P_16_*t*. (**b**) Size exclusion chromatograms of P188 and E_182_P_16_*t*.

**
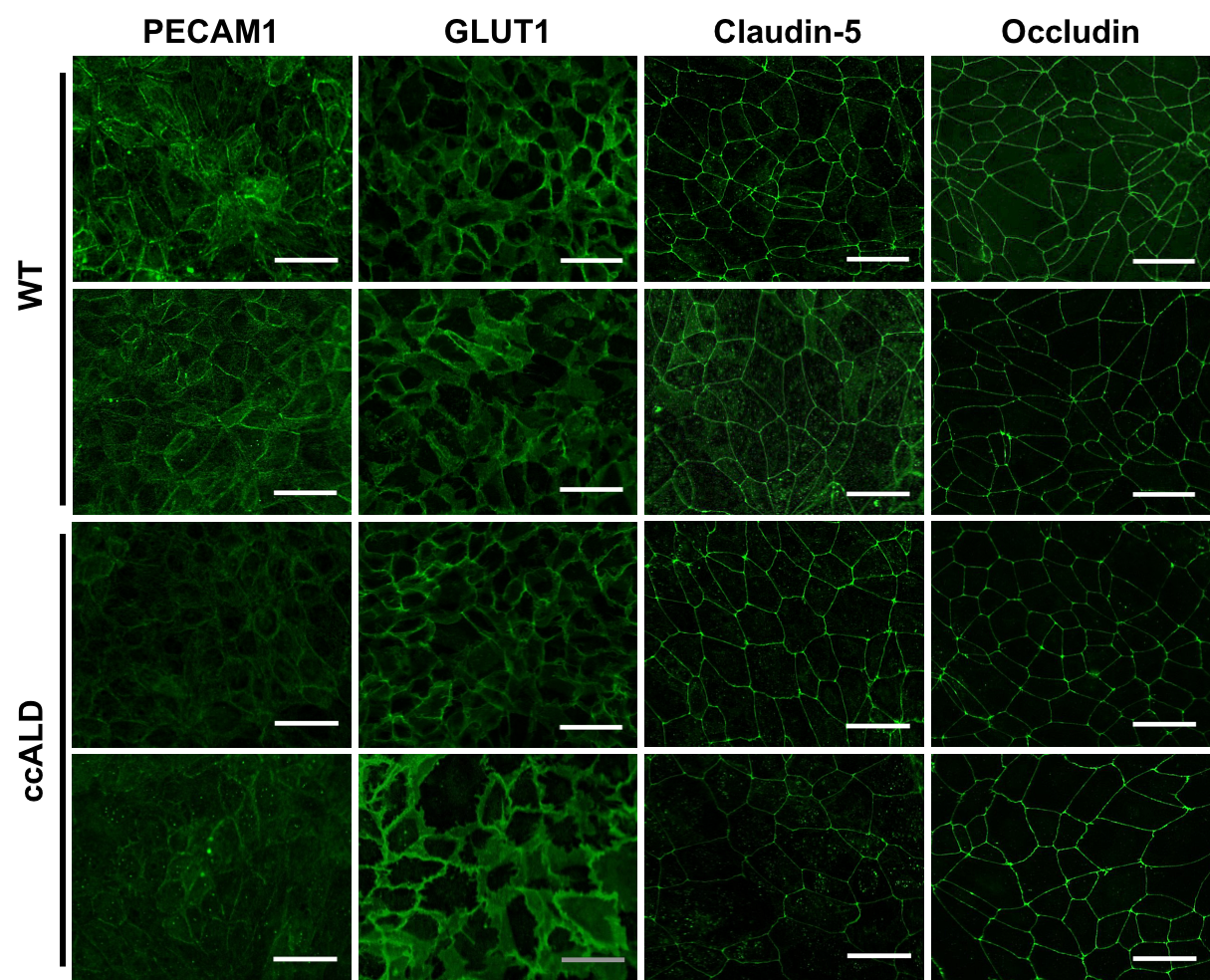
**

**Figure S2. Representative immunocytochemistry images of iBMEC lines not shown in main manuscript.** All iBMECs express the requisite endothelial, tight junction, and BBB markers by immunocytochemistry. iBMECs from ccALD patients and WT controls express PECAM1*,* GLUT1*,* claudin-5, and occludin. Top to bottom: WT2, WT3, ccALD1, ccALD3. Scale bar = 50 µm.

**
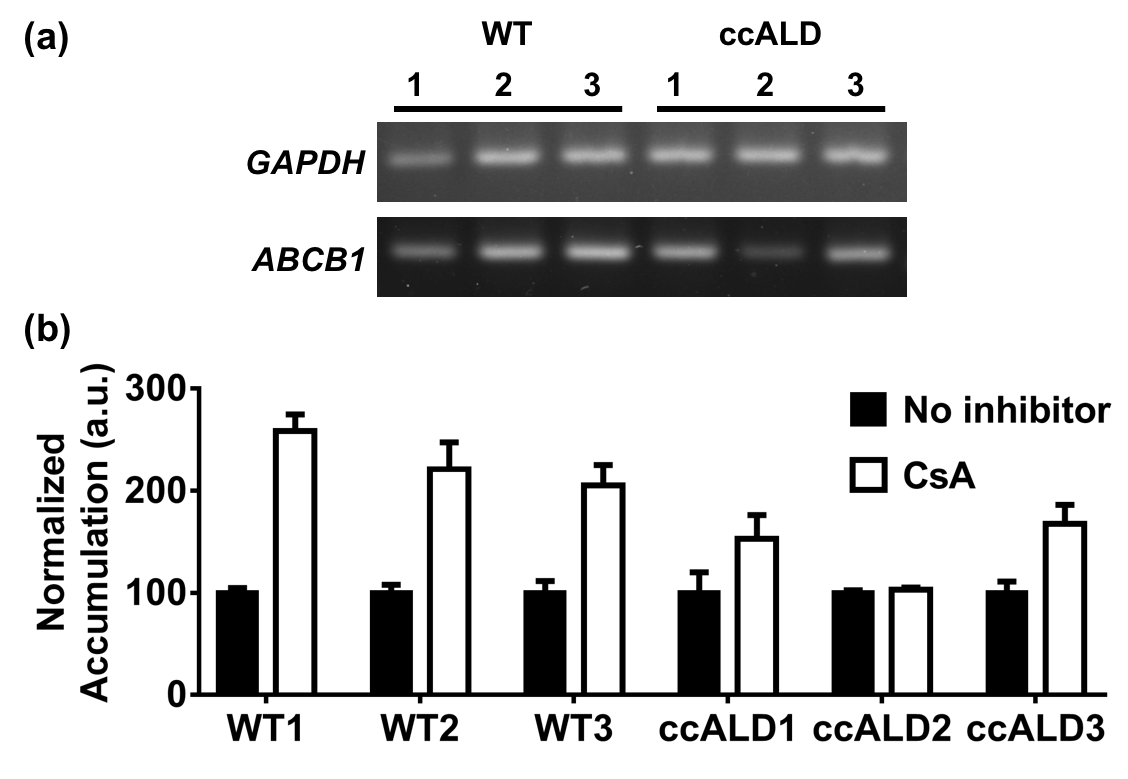
**

**Figure S3. P-glycoprotein (P-gp) expression and function.** (**a**) All WT- and ccALD-iBMECs express *ABCB1*, which codes for BMEC-specific efflux transporter P-gp. (**b**) P-gp function was assessed with Rhodamine 123 accumulation assay, which showed functional P-gp for all iBMECs except ccALD2-iBMECs. Differences in normalized accumulation between no inhibitor and CsA inhibited samples are statistically significant (p < 0.05) for all cell lines with the exception of ccALD2-iBMECs. Fluorescence intensity is normalized by cell density, and accumulation is independently normalized to the corresponding control (no inhibitor). Four biological replicates used (n = 4).


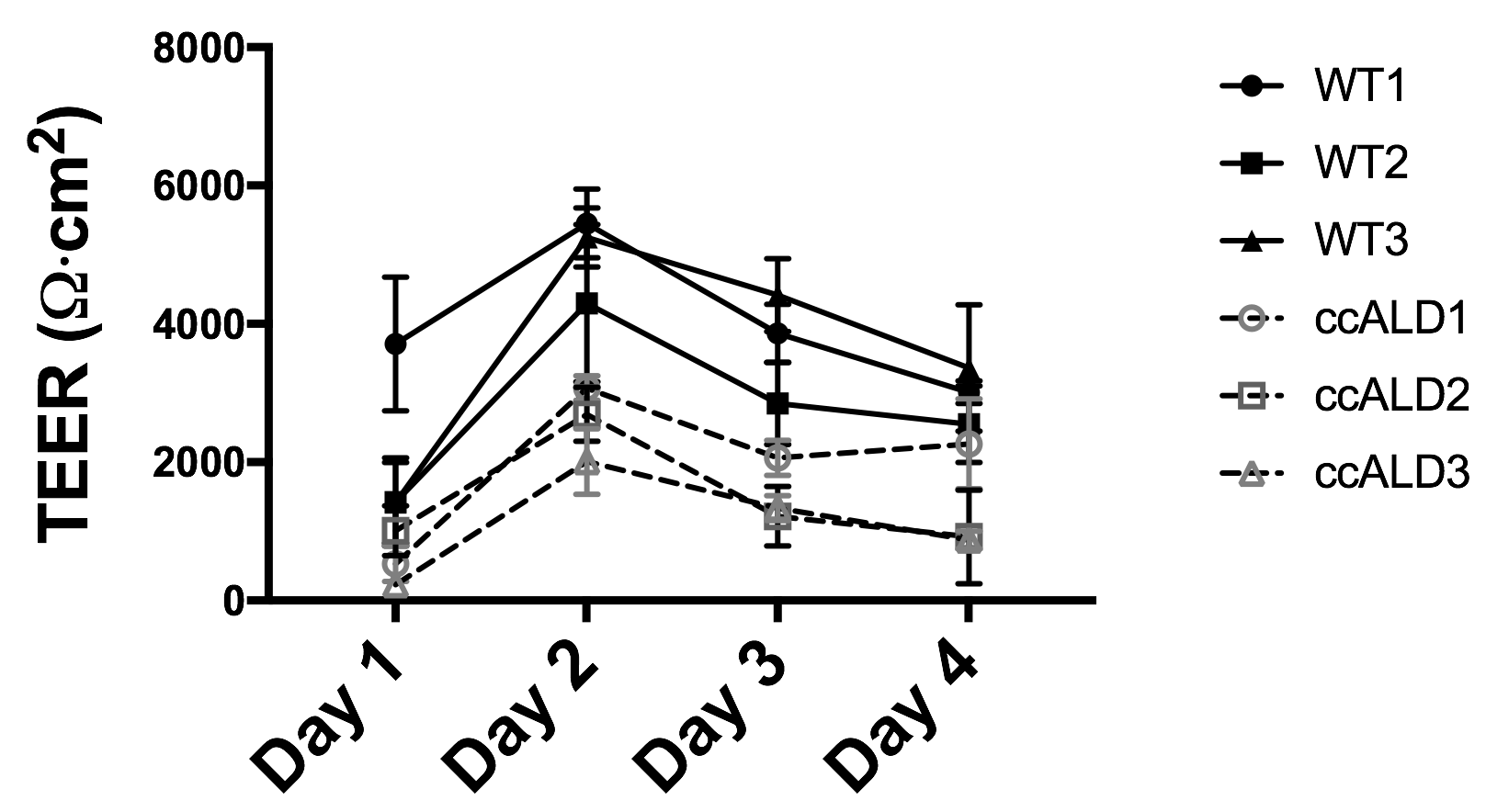


**Figure S4. TEER measurements for individual cell lines.** Trans-endothelial electrical resistance (TEER) is lower for the ccALD-iBMECs compared to the WT-iBMECs at all days measured. Data compiled from three independent experiments with three biological replicates for each cell line (n = 9).

**
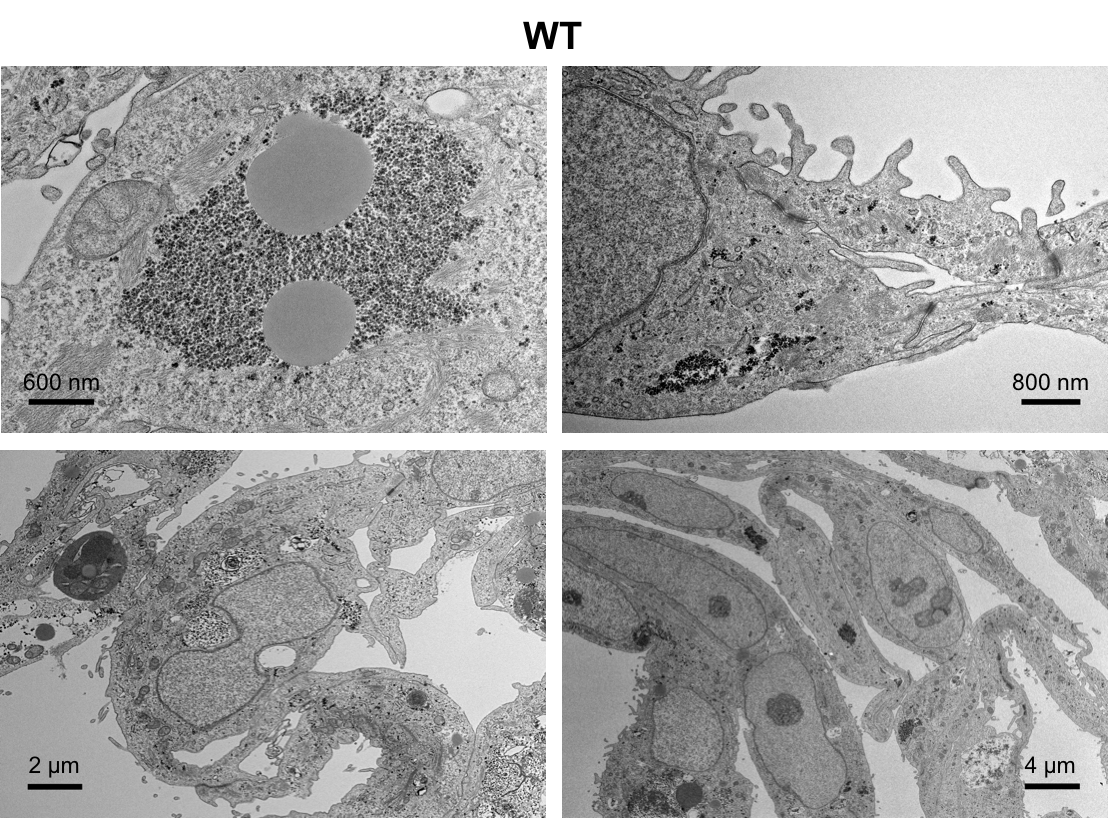

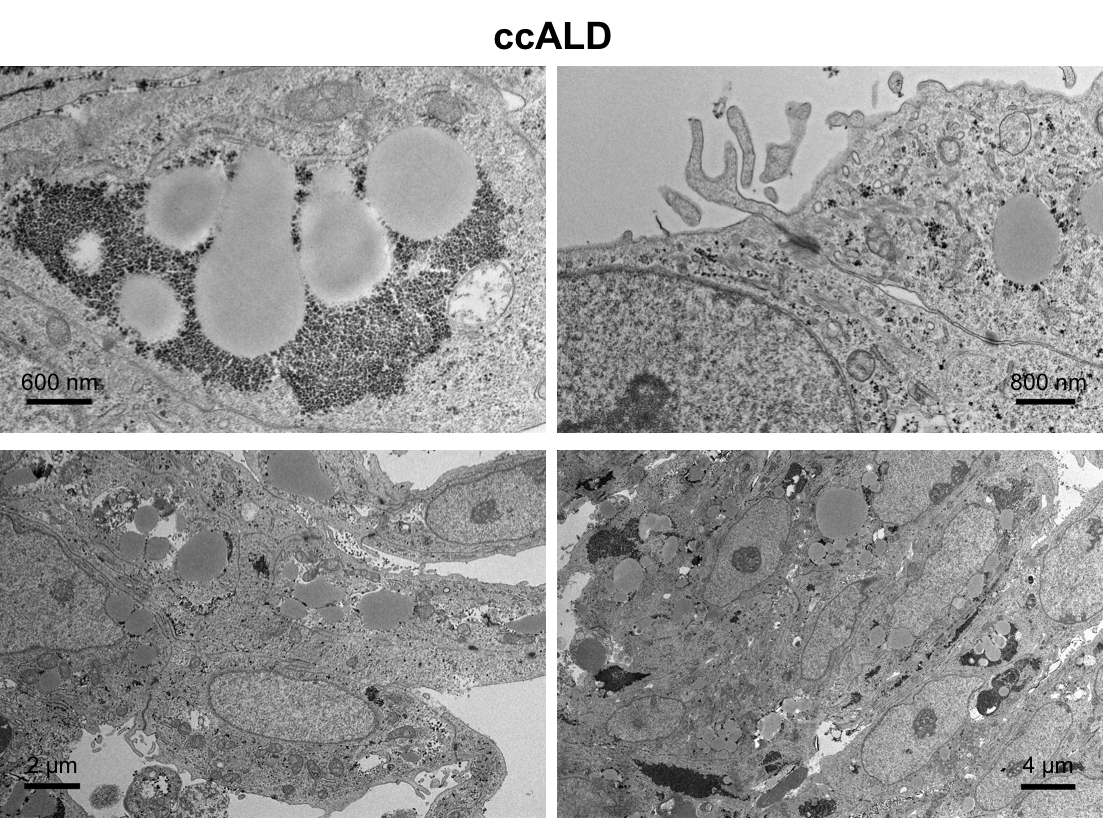
**

**Figure S5. Additional representative TEM images.** TEM of WT1-iBMECs (above) and ccALD3-iBMECs (below) at varying magnifications showing increased lipid droplet accumulation in ccALD-iBMECs.

**
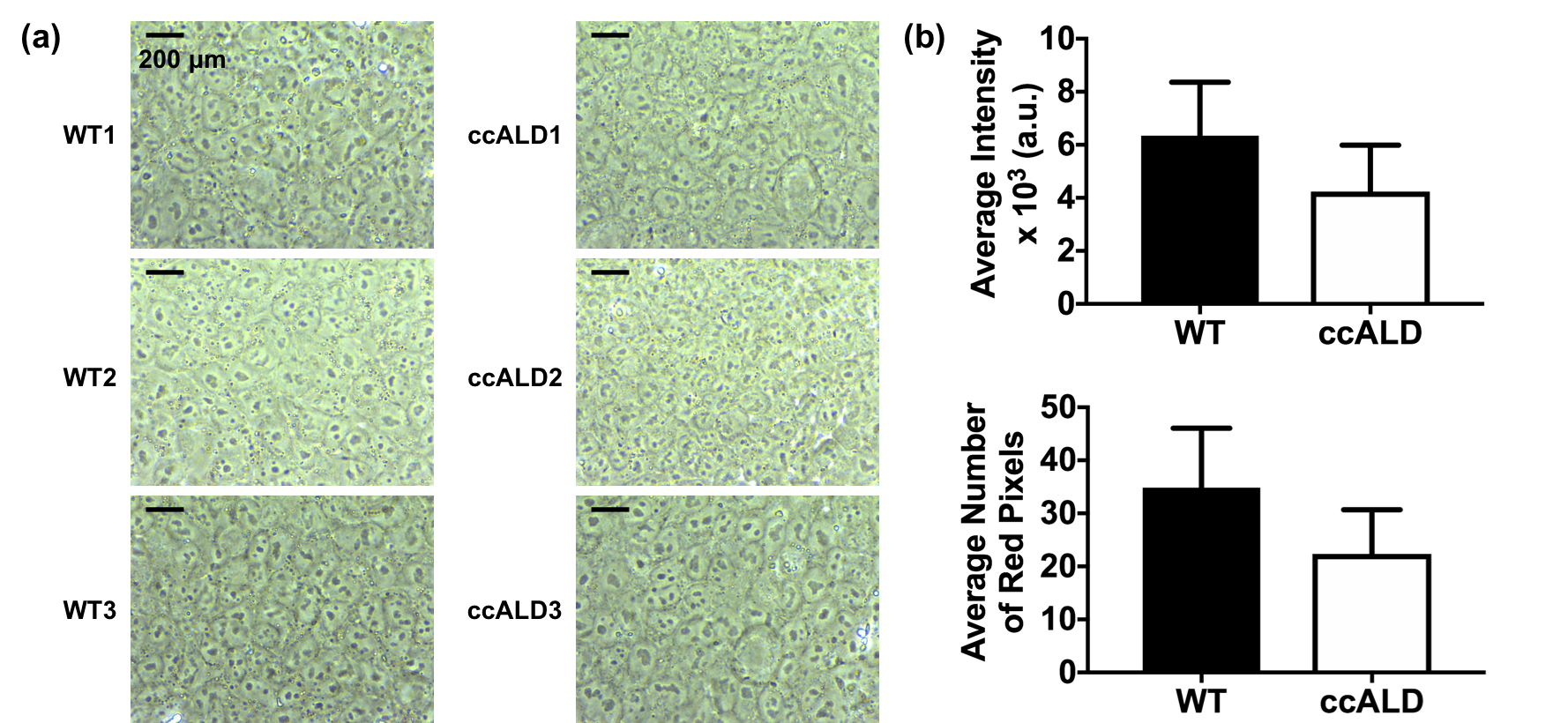
**

**Figure S6. Oil-Red-O staining and quantification of WT and ccALD-iPSCs.** (**a**) Oil-Red-O staining of WT and ccALD-iPSCs show little to no lipid droplet accumulation. (**b**) Quantification of intensity and number of red pixels in images of Oil-Red-O stained iPSCs show no difference in lipid droplet accumulation in ccALD-iPSCs compared to WT-iPSCs. All cell lines were used for quantification with two biological replicates each (n=6).


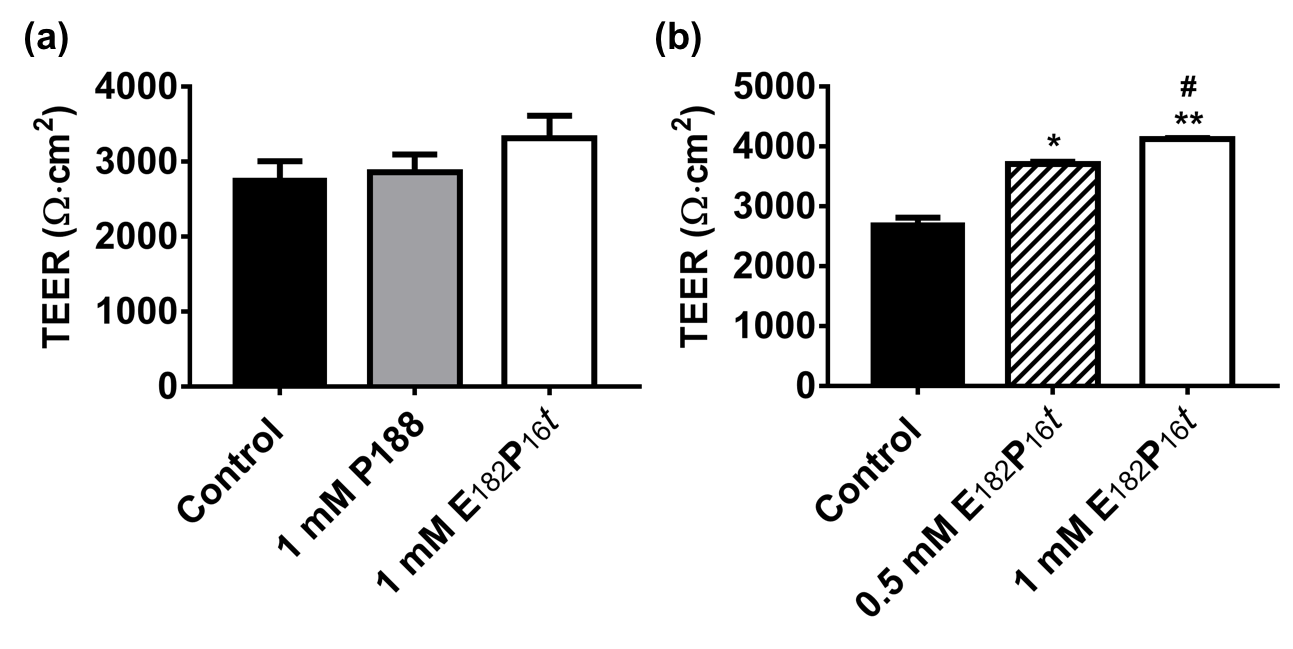


**Figure S7. Timing and dosage effect of polymer treatment.** (**a**) Addition of 1 mM P188 or E_182_P_16_*t* on day 9 of the differentiation protocol had a non-significant effect on TEER of the ccALD3-iBMECs. However, a slight increase in TEER upon treatment with 1 mM E_182_P_16_*t* was observed. Data compiled from three independent experiments with three biological replicates each (n = 9). (**b**) Maximum TEER of ccALD3-iBMECs treated 1 mM E_182_P_16_*t* on day 3 of the differentiation protocol is higher than ccALD3-iBMECs treated with 0.5 mM E_182_P_16_*t* on day 3, signifying that treatment efficacy is concentration dependent. Data from three biological replicates (n = 3). * p < 0.005, ** p < 0.0005 with respect to control. # p < 0.001 with respect to 0.5 mM E_182_P_16_*t* condition.
